# Synthetic circuits that process multiple light and chemical signal inputs

**DOI:** 10.1186/s12918-016-0384-y

**Published:** 2017-01-19

**Authors:** Lizhong Liu, Wei Huang, Jian-Dong Huang

**Affiliations:** 1 School of Biomedical Sciences, Li Ka Shing Faculty of Medicine, University of Hong Kong, Pok Fu Lam, Hong Kong, People’s Republic of China; 2 Shenzhen Institute of Research and Innovation, University of Hong Kong, Shenzhen, 518057 People’s Republic of China; 3 The Centre for Synthetic Biology Engineering Research, Shenzhen Institutes of Advanced Technology, Shenzhen, 518055 People’s Republic of China; 4 Department of Biology, Shenzhen Key Laboratory of Cell Microenvironment, South University of Science and Technology of China, Shenzhen, 518055 People’s Republic of China

**Keywords:** Synthetic circuit, Multi-input, Signal integration, Light-inducible

## Abstract

**Background:**

Multi-signal processing circuits are essential for rational design of sophisticated synthetic systems with good controllability and modularity, therefore, enable construction of high-level networks. Moreover, light-inducible systems provide fast and reversible means for spatiotemporal control of gene expression.

**Results:**

Here, in HEK 293 cells, we present combinatory genetic circuits responding to light and chemical signals, simultaneously. We first constructed a dual input circuit converting different light intensities into varying of the sensitivity of the promoter to a chemical inducer (doxycycline). Next, we generated a ternary input circuit, which responded to light, doxycycline and cumate. This circuit allowed us to use different combinations of blue light and the two chemical inducers to generate gradual output values over two orders of magnitude.

**Conclusions:**

Overall, in this study, we devise genetic circuits sensing and processing light and chemical inducers. Our work may provide insights into bio-computation and fine-tuning expression of the transgene.

**Electronic supplementary material:**

The online version of this article (doi:10.1186/s12918-016-0384-y) contains supplementary material, which is available to authorized users.

## Background

Synthetic biology adopts the concepts of engineering and computational science into biological systems, aiming to generate artificial genetic circuits and systems with desirable functions. It offers a promising way to address global challenges, for example, clean energy, environment restoration, and increasing medical needs. During the past decade, a remarkable development of synthetic biology has been achieved. Various synthetic devices and systems have been established, including biological oscillators, switches, counters, as well as logic gates [1–4]. However, it is still challenging to generate complex synthetic systems with good controllability and programmability. For instance, like electronic or mechanical systems that can be fine-tuned by various inputs and produce predictable outputs. Therefore, one can program the systems to act in a desirable way by altering input information. Development and characterization of standard modules, which sense and convert multiple input signals into cellular responses will help address the challenges [5, 6].

In natural biological systems, multi-signal processing is a fundamental aspect. For example, the bow-tie (also called hourglass) architecture, which refers to systems that receive a diversity of inputs and convert the input signals through an intermediate “core”, and finally generate a variety of outputs. Since the intermediate “core” is composed of relatively few universal components, the overall structure of the system resembles a bow-tie or hourglass [7]. For instance, in metabolic networks, multiple input nutrients are converted into multiple biomass components by a small number of mediator factors [7]. Previous work suggests that the recurrence of bow-tie architecture in various biological systems indicates its significance on enhancing the robustness of the biological systems [8]. In the counterpart electronic systems, modules for multi-input integration are also widely used, for example, a module called “digital-to-analog” converter (DAC) is commonly used in audio or video devices for converting multiple digital-input signals into the analog output signals [9].

Previous work reported some chemically-inducible expression systems in mammalian cells [10, 11]. Recently, Optogenetics has demonstrated that light is an ideal source of signal for spatiotemporal control of gene expression [12–15]. The combination of chemical inducer and light inducer, for example by generating chimeric promoters that consist chemical-responsive and light-responsive elements, can achieve spatial and stringent control of transgenes [16]. Using light as inducer can avoid drawbacks of using chemical inducers. For instance, the chemical inducers are needed to be transported into cells by passive or active manner before they encounter the sensors, which causes a delay of target gene expression. The delay may lead to undesirable cell-to-cell variation. However, using light does not result in this problem. In addition, recent work demonstrated that light can be used as a communication signal between the computer and modified *E. coli* cells [17, 18]. Connecting synthetic biology systems with a computer, and then monitor and control the behaviors of the circuits by a computer program can tremendously increase programmability of synthetic systems.

In this study, we first developed a 2-input circuit that exhibited different sensitivity to doxycycline (Dox) upon different doses of blue light illumination. Specifically, a blue light-inducible system, called LightOn system [14], was used to control the expression level of a transcriptional repressor TetR. A reporter GFP was driven by TetR-repressible promoter. The repression of TetR can be relieved by adding Dox. Therefore, light and Dox acted as inducers of this circuit. Next, we generated a 3-input circuit for conversion of the binary input sequence, consisting of light and chemicals, into graded output promoter activities. Specifically, this circuit was composed of a cumate-inducible promoter driving a modified rtTA (hereafter, rtTAm) [19], a light-inducible promoter driving the TetR co-repression peptides (hereafter, TCP) [20]. TCP-rtTAm complex activates the output TRE3G promoter. Therefore, Light-inducible system and cumate-switch system form an AND-gate. On the other hand, Dox also can trigger the DNA-binding of rtTAm. Therefore, Dox-inducible system and cumate-switch system also compose an AND-gate. Previous work suggests that short peptide inducer may be less efficient than Dox [21]. Moreover, it has been reported that the peptide competes with Dox for the tc-binding pocket of TetR [20]. Thereby, the potency of TCP fusion protein might be much lower than Dox as rtTAm inducer, and the presence of TCP fusion protein could inhibit Dox inducing ability.

## Results

### The dual input circuit converting illumination dose into sensitivity variations of a promoter to Dox

**Fig. 1:**
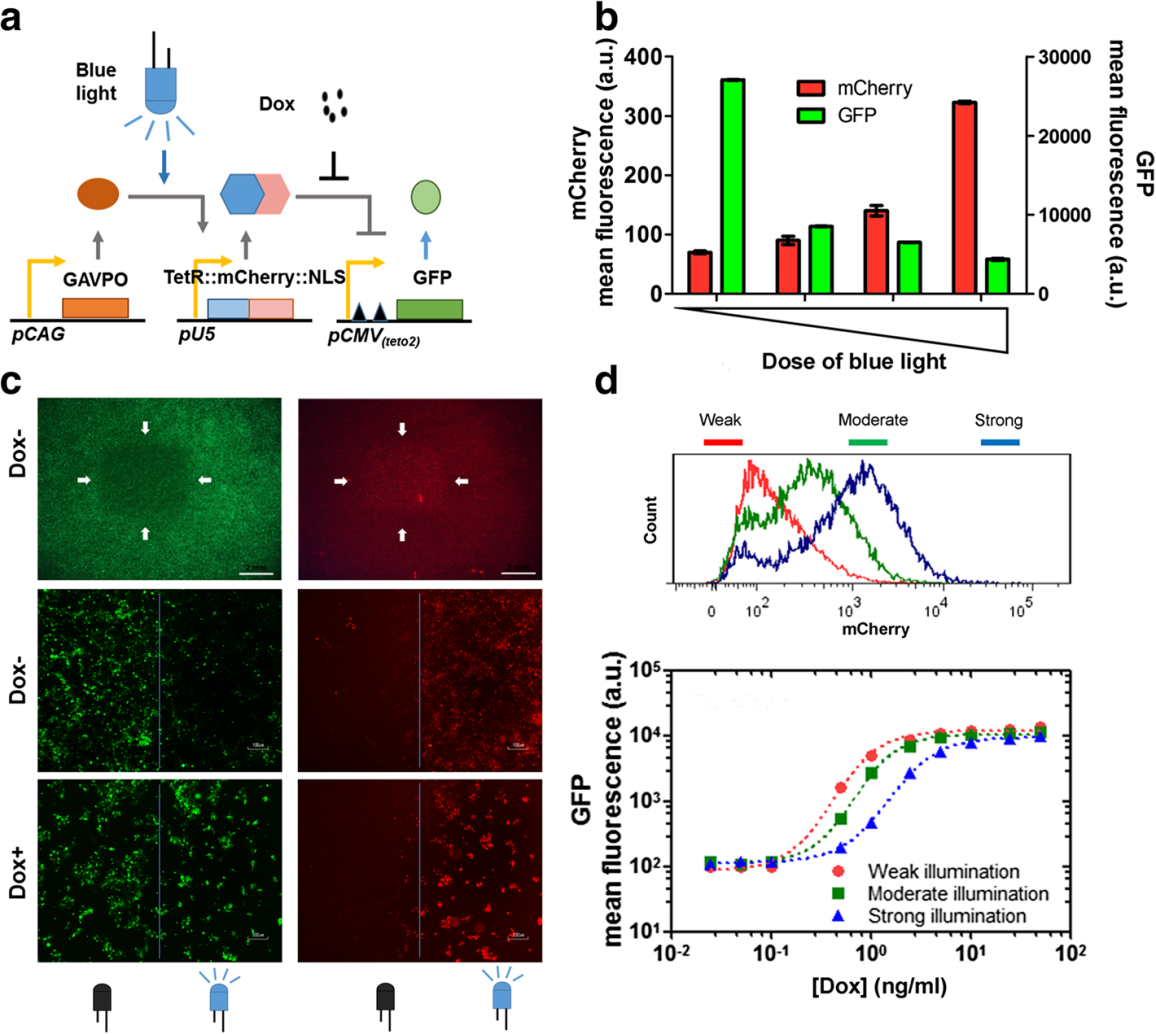
Light-switchable synthetic circuit with tunable activation threshold and spatial resolution. (**a**) Schematic diagram of the circuit. The CAG promoter is constitutively expressing the photoactive transactivator GAVPO. Upon blue light illumination GAVPO forms a homodimer, which then initiates the transcription of TetR::mCherry::NLS from the pU5 promoter. GFP is under the control of TetR::mCherry::NLS-repressible promoter CMV(tetO2). Dox can release the repression. (**b**) Cells were illuminated with blue light (1.25 W m^−2^) for different durations (dark, 10 min, 30 min, and 3 h) in the absence of Dox, followed by 24 h incubation in dark. Data are presented as mean ± SEM (*n* = 3). (**c**) A square area, which is indicated by white arrows in the upper panel, was illuminated by blue light (1.25 W m^−2^) for 24 h. In the middle and low panel, the boundary between illuminated and dark area was indicated by the blue line. The right part of each picture is the illuminated area, while the left part is the dark area. Cells shown in the low panel were treated with 1 μg/ml of Dox. Scale bar is 2 mm in the upper panel, and 100 μm in the middle and low panel. (**d**) Cells with different levels of TetR::mCherry::NLS differentially responded to Dox. The upper panel shows the mCherry intensity of the cells illuminated with blue light (1.25 W m^−2^) for 1 h (weak, red line), 5 h (moderate, green line), or 20 h (strong, blue line), respectively. The lower panel shows GFP intensity of cells treated with different concentration of Dox after illumination. The data are presented as mean ± SEM (*n* = 3). Data are fitted to a modified Hill equation (dashed lines). The EC50s for the three curves are 2.70 ± 0.15 ng/ml (red), 4.74 ± 0.13 ng/ml (green), and 35.81 ± 1.03 ng/ml (blue). The Hill coefficients for the three curves are 1.81 ± 0.15 (red), 1.77 ± 0.11 (green), and 1.67 ± 0.06 (blue)

In this circuit, LightOn system was used to control the expression of TetR. And a reporter GFP was driven by the CMV(tetO2) promoter (Life Technologies, T-REx system, and Additional file 1: Supplementary note) containing two copies of tet operator 2 (Fig. 1a). The LightOn system comprises a synthetic photoactive transactivator GAVPO and its cognate synthetic promoter U5 [14]. GAVPO monomers form a homodimer upon blue light illumination. The GAVPO dimer then binds to the UAS_G_ element in the U5 promoter to recruit general transcription factors and coactivators to bind to the U5 promoter.

Our data indicated the expression level of the TetR::mCherry::NLS fusion could be tuned by adjusting the exposure of blue light (Fig. 1b-d). We examined the spatial resolution of this circuit. Specifically, we illuminated a small square area of the dish, while the other area of the dish was kept in dark. The result showed that cells in the illuminated area were TetR::mCherry::NLS positive and GFP low, while cells in the adjacent dark area were TetR::mCherry::NLS negative and GFP high. The light-induced TetR::mCherry::NLS suppressed the expression of GFP, and addition of Dox relieved this repression (Fig. 1c). Furthermore, we illuminated the cells with different doses of blue light before Dox treatment. Cells exposed to various amounts of light showed different activation thresholds of Dox induction. The increase in the level of the repressor resulted in an increase in the Dox threshold. Whilst, the dynamic range of the promoter was not affected (Fig. 1d).

### Design and construction of a multi-input circuit for conversion of light and chemical binary information into different promoter activities

**Fig. 2:**
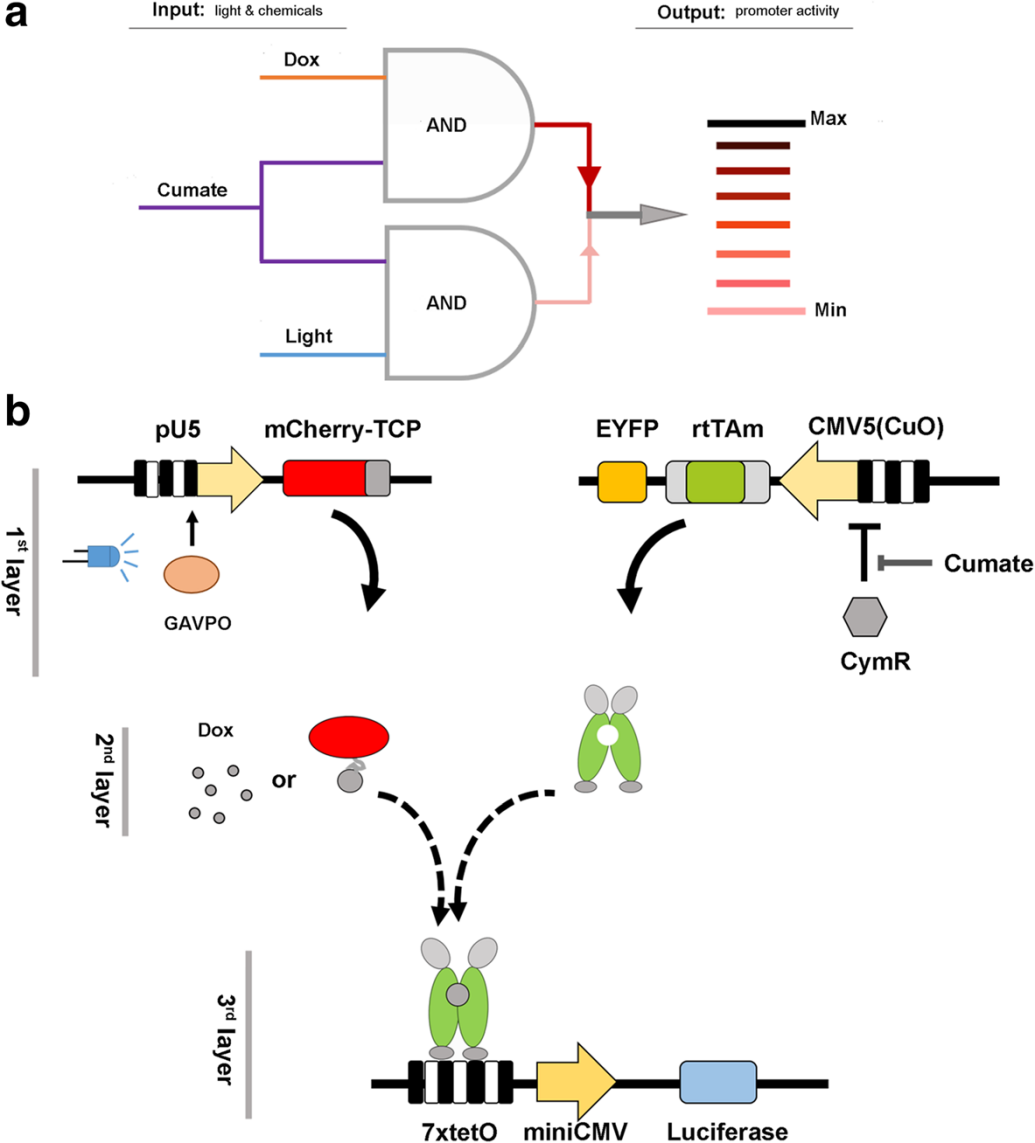
The design of the 3-input circuit. (**a**) Illustration of the conversion of input signals including Dox, cumate and light into graded output signals, which are promoter activities. The system consists of three inducible systems formed Boolean logic gates. (**b**) The scheme of the circuit. It can be divided into three layers. In the first layer, LightOn system controls the expression of mCherry-TCP fusion; cumate-switch system controls the expression of rtTAm (co-expressed with EYFP linked by 2A peptide). In the second layer, Dox or mCherry-TCP fusion protein can serve as an inducer to trigger the binding of rtTAm to the cognate TRE3G promoter. In the third layer, rtTAm serves as transcription activator to initiate the transcription of luciferase from TRE3G promoter

Next, we attempted to design and construct a circuit for integration and conversion binary combinations of light and chemical signals into graded output values (Fig. 2a).

The circuit consists of two AND-gates that response to blue light, cumate and Dox, respectively (Fig. 2a, b). However, the output strengths of the two AND-gates were not equivalent. The addition of saturated cumate and Dox resulted in strong output while the addition of cumate together with light illumination led to moderate output (Fig. 5).

The components composing the circuit are as following: LightOn system which was introduced above; the cumate-inducible system consists of a transcription repressor CymR and the repressible promoter CMV5CuO promoter (pCMV5CuO). CymR binds to the operator sequence (CuO) downstream of a strong promoter CMV5 and inhibits transcription. The addition of a cumate relieve the repression by CymR [19]; and a TetR co-repression peptide (TCP) -inducible system. Specifically, the mCherry-TCP fusion (Additional file 1: Supplementary note) can aid in DNA-binding of rtTAm. The rtTAm is a chimeric protein composed of a reverse TetR variant and 3 copies of VP16 activation domain (Additional file 1: Supplementary note). The TCP binds to the tc-binding pocket of the reverse TetR variant and triggers allosteric conformational change in the reverse TetR variant, leading to binding of the latter to its cognate DNA [20]. The responsive TRE3G promoter contains seven repeats of tet operator site (tetO) upstream of a CMV minimal promoter (Fig. 2b).

The circuit can be divided into three layers. The first layer receives light and cumate signals. In this layer. constitutively expressed GAVOP (it does not appear in the scheme) activates expression of mCherry-TCP upon blue light illumination. Constitutively expressed CymR (it does not appear in the scheme) suppresses the expression of rtTAm. Cumate is required to switch on the expression of rtTAm. The second layer is the information integration node. Specifically, the rtTAm, representing the presence of cumate, and mCherry-TCP, representing the presence of blue light, interact and form a protein complex, which can activate the output promoter. On the other hand, Dox also can aid binding of rtTAm to output promoter. The third layer is the responding (output) node, in which there is a luciferase gene driven by the TRE3G promoter (Fig. 2b).

### Characterization of the ternary input circuit

We first characterized each inducible expression node, separately. And identified the saturation dose for each inducer. Then we characterized the complete circuit.

**Fig. 3:**
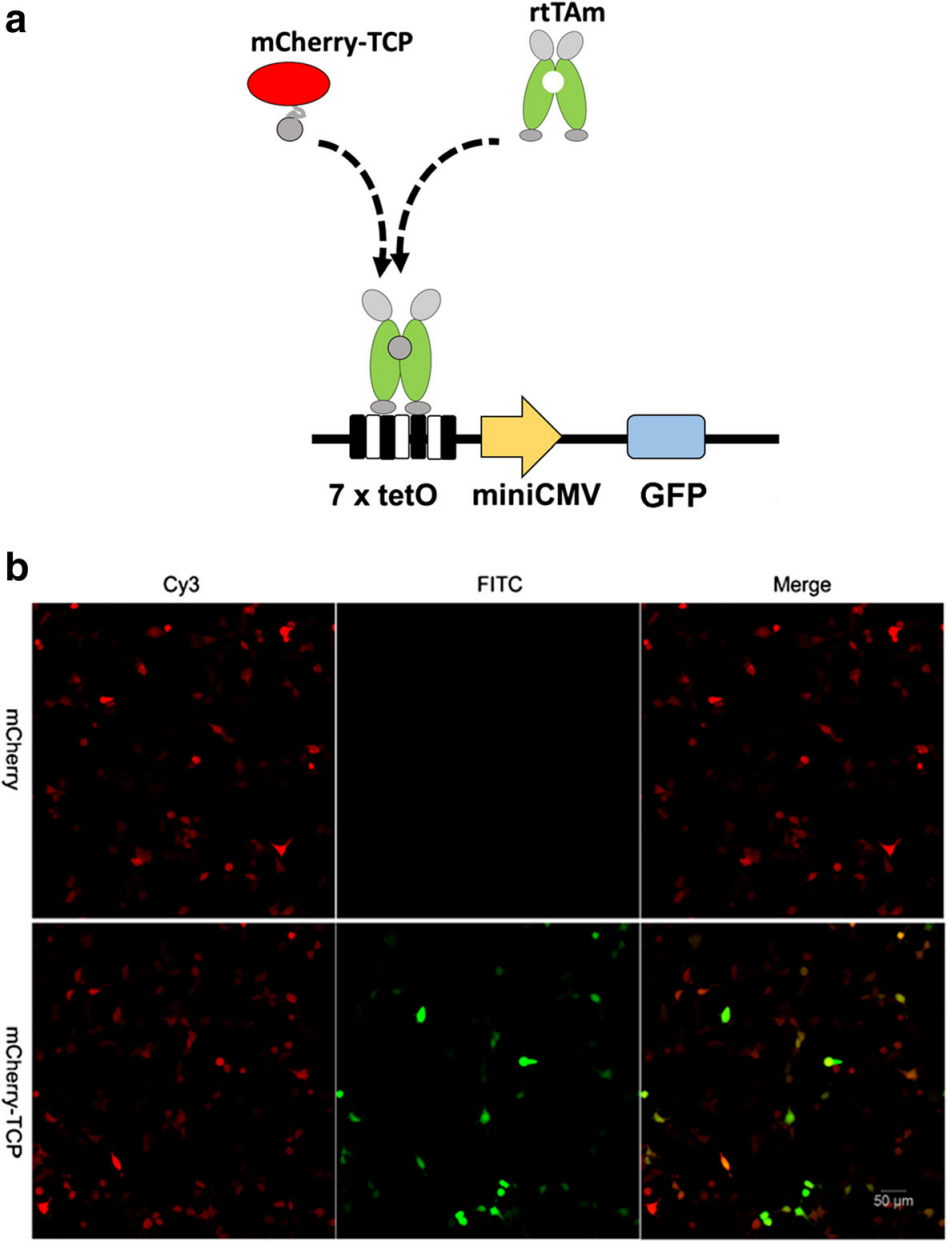
TCP induces rtTAm. (**a**) Scheme of TCP induced rtTAm binding to TRE3G promoter. Constitutively expressed mCherry-TCP and rtTAm (both driven by CMV promoter) interact with each other, and then the complex bind to the tetO elements within TRE3G promoter (consist of 7 × tetO elements and a minimal CMV promoter). A GFP gene is placed downstream of TRE3G promoter. (**b**) mCherry-TCP induced the rtTAm-dependent expression of GFP. GFP-positive cells were observed in cells co-transfected with mCherry-TCP and rtTAm coding plasmids, but not in cells co-transfected with mCherry and rtTAm coding plasmids. The scale bar is 50 μm

To our knowledge, there is no demonstration of induction of rtTA (in our case, it is rtTAm) by TCP in mammalian cells, to date. We first attempted to test whether intracellularly expressed TCP fusion protein, i.e., mCherry-TCP fusion, could induce rtTAm-dependent expression from the TRE3G promoter. To this end, we used CMV promoter to control the expression of mCherry-TCP and rtTAm. And used TRE3G promoter to drive a GFP gene (Fig. 3a). We introduced the circuit DNA into HEK293 cells by transfection and observed the transfected cells by fluorescent microscope, 72 h after transfection. In the control group, the mCherry-TCP fusion was replaced by mCherry. We observed GFP signal in the mCherry-TCP cells but not in the mCherry cells (Fig. 3b and Additional file 2: Figure S1).

**Fig. 4:**
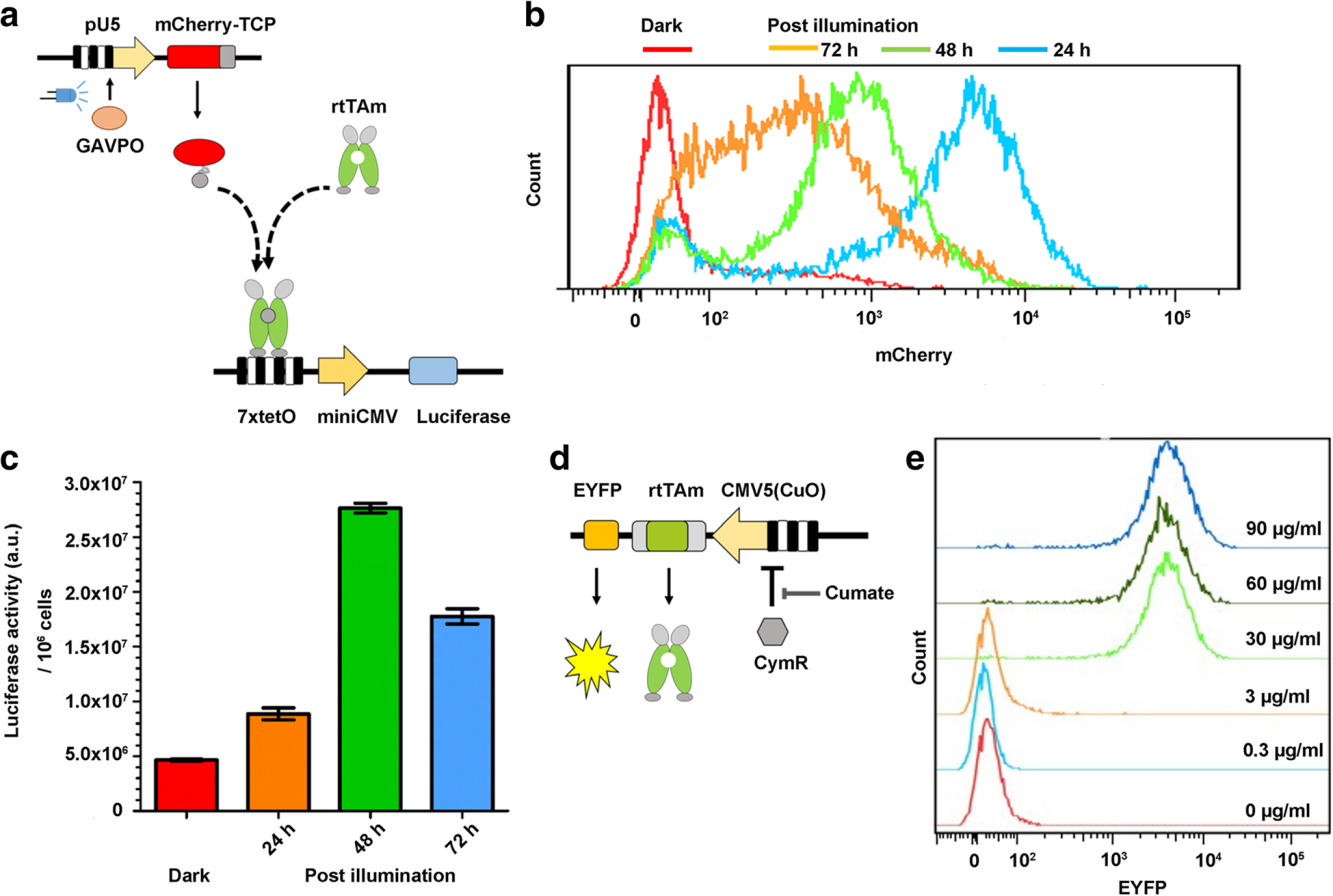
(**a**) Scheme of the light-induced expression of mCherry-TCP, which interacts with rtTAm, and then the complex binds to tetO elements in TRE3G promoter. (**b**) Light-induced expression of mCherry-TCP. One group of cells was not illuminated by blue LED (Red line). The rest three groups of cells were illuminated by blue LED (1.25 W m^−2^) for 24 h followed by further 48 h (72 h in total, orange line), 24 h (48 h in total, green line), or 0 h (24 h in total, blue line) incubation in a dark environment. (**c**) mCherry-TCP induced expression of luciferase. The luciferase expression levels of the above cells were examined. “0 h” represents cells without illumination, “24 h” represents cells illuminated for 24 h without further incubation in the dark, “48 h” represents cells illuminated for 24 h then incubated in dark for further 24 h, “72 h” represents cells illuminated for 24 h then incubated in dark for 48 h. The data are presented as mean ± SEM (*n* = 6). (**d**) Scheme showing the cumate-inducible expression of rtTAm and EYFP. (**e**) Cumate-inducible expression of EYFP examined by flow cytometry. The corresponding cumate concentrations are indicated

To characterize the LightOn system regulated expression of mCherry-TCP, we generated a stable cell line integrated with a modified circuit without expression of CymR. However, the other components were the same as the complete circuit (Fig. 4a). We illuminated the cells with blue LED (1.25 W m^−2^) for 24 h and then put the cells back in a dark environment for 0 h, 24 h, or 48 h (the total incubation time was 24 h, 48 h or 72 h, respectively). Next, we examined the mCherry-TCP expression levels by flow cytometry (Fig. 4b). At the meantime, we examined luciferase expression induced by mCherry-TCP. The data suggested that after 24 h illumination, the expression of mCherry-TCP reached the highest level, and then it start to decrease. However, the maximum expression level of luciferase was observed 48 h after the illumination started (Fig. 4c). In another experiment, we examined the kinetics of the circuit at earlier phases after illumination with constitutively expressed rtTAm. The results show that 10 h after illumination a moderate increase of luciferase activity was detected (Additional file 3: Figure S2A). And the response of the circuit to blue light was slower than the response to Dox (Additional file 3: Figure S2B).

Also, we characterized the cumate-inducible node in the circuit (Fig. 4d). We varied cumate concentrations, and then examined the expression of EYFP. The data suggested that addition of 30 μg/ml of cumate in the medium was enough to produce the maximal expression level of rtTAm and EYFP from pCMV5CuO (Fig. 4e). The kinetics of the circuit responding to cumate induction with constitutive mCherry-TCP expression was also characterized. In the experiment, the cells were continuously illuminated, meanwhile were treated with 30 μg/ml of cumate for different time durations. 10 h after cumate addition, a moderate increase of luciferase activity was detected (Additional file 3: Figure S2C).

**Fig. 5:**
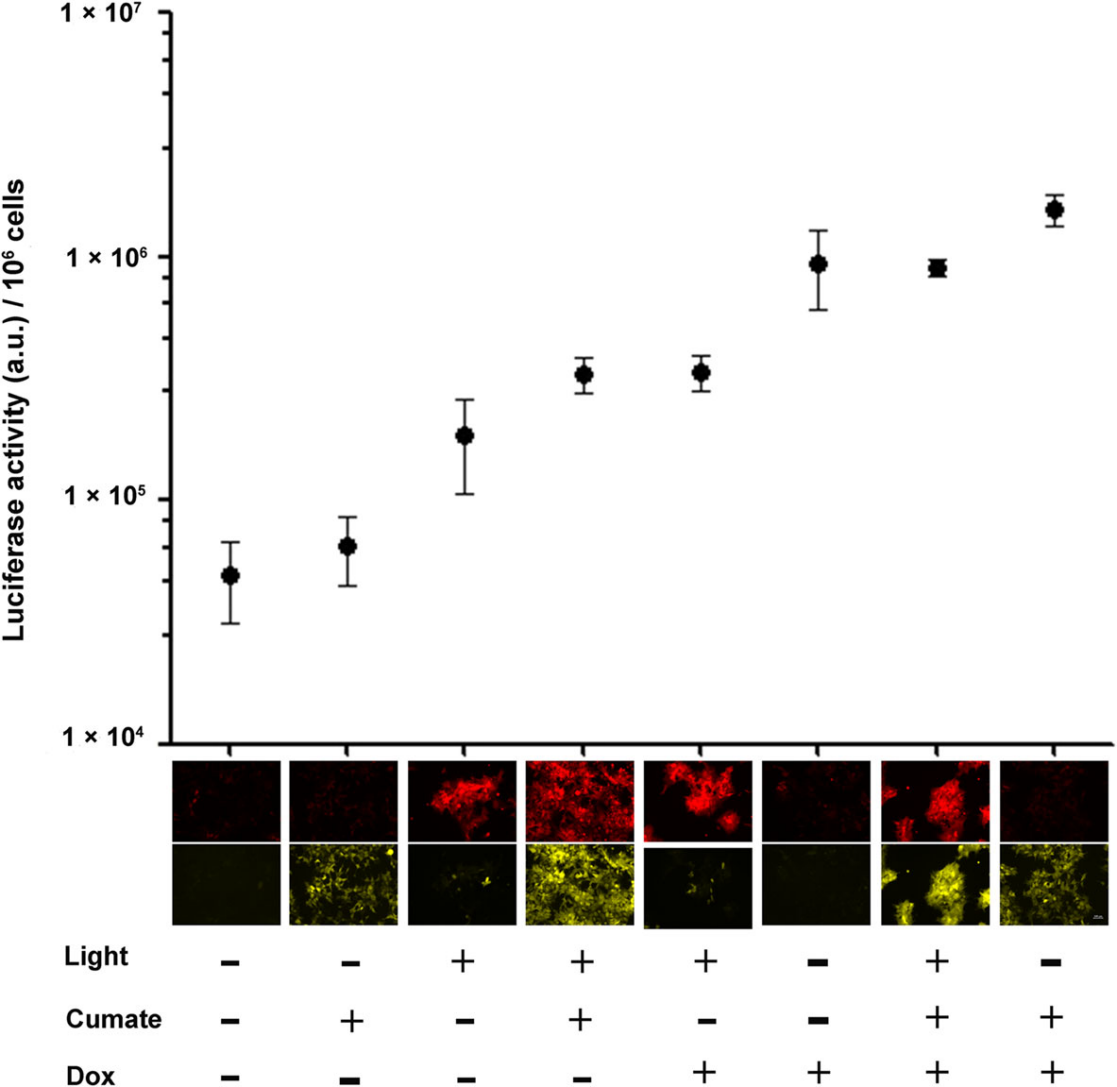
Characterization of the complete 3-input circuit. Microscopy images, showing the expression of mCherry-TCP (Cy3 filter) and EYFP (YFP filter) of cells treated with all eight combinations of three inducers, are presented beneath the corresponding values of luciferase activity. Scale bar in the last image is 100 μl. All the images were taken at the same magnification. The x-axis indicates the specific treatments. “+” represents illumination with blue LED (1.25 W m^−2^) for 24 h then incubated in the dark for another 24 h, treatment of 1 μg/ml of Dox, or treatment of 30 μg/ml of cumate, respectively. “-” represents no corresponding treatments. Expression of luciferase of the cells treated with various input signals combinations was examined. The y-axis shows luciferase activity. Data are presented as mean ± SD (*n* = 6)

Finally, we characterized the complete circuit. We applied all the eight combinations of inputs to the cells and measured the output values, i.e., the luciferase activities. Expression levels of mCherry-TCP and rtTAm (indicated by EYFP) were also examined. The data showed that the circuit responded to different input combinations and generated different output values, which evenly distributed in a range of two orders of magnitude. The output signal induced by TCP was weaker than the signal induced by Dox. In agreement with previous work [20], TCP inhibited the rtTAm-binding of Dox, which might explain that the input combination of “Light +, Cumate +, Dox +” induced lower luciferase level than the combination of “Light **-**, Cumate +, Dox+” (Fig. 5). We also introduced a conditional positive feedback loop into the third layer. A trans-activator, i.e., tTA was placed downstream of TRE3G promoter. The binding of tTA to TRE3G promoter can be blocked by Dox, but not by TCP (data not shown). The result suggested that the conditional positive feedback circuit responded to the input combinations similarly as the circuit without feedback (Additional file 4: Figure S3).

## Discussion

We observed leaky expression in the 3-input circuit, specifically, the leakage of rtTAm and mCherry-TCP was observed (Fig. 5). The leaky expression usually causes undesired basal expression of regulated proteins in the absence of the respective inducers and results in compromise of the circuits [22, 23]. However, the leaky expression here might lead to the differential responses of the circuit to individual input combinations, which is not completely undesirable. It is possible that due to the leaky expression of rtTAm and mCherry-TCP, each inducer, i.e., light, cumate or Dox, exhibited different effects to the system. For instance, when cumate was added in the medium, the elevated rtTAm level could enhance leaky expression of TRE3G promoter, in the absence of the other two inducers; when the cells were illuminated, increased mCherry-TCP interacted with leaky rtTAm, and resulted in higher rtTAm-dependent expression of luciferase. Previous study demonstrated the way to fine-tune basal and/or maximal expression of LightOn system [24], which provides insights for the future modification of the circuit, especially when stringent control of a specific promoter is needed. Similarly, lower leaky expression level would be achieved by modifying CMV5(CuO) promoter using another weaker enhancer element to replace the strong CMV enhancer or increase the level of CymR by using a stronger promoter.

Our circuits can be used as building blocks in a synthetic programmable system. For instance, it can be utilized in a synthetic bow-tie structure, which is designed to sense and convert multiple input signals into the adjustment of master regulatory factors, for cell fate decision or cell cycle control. Recently, researchers from another group demonstrated optically programmed gene expression control [17, 18]. In their work, the light sequence generated by computer were used to control dynamics of synthetic circuits. Our circuit also can be modified to respond to a computer-generated sequence of light of different wavelengths. For instance, a red- or far-red light-responsive system [25], can be used to replace the cumate-inducible part in the current circuit. Thereby it may allow us to convert digital codes generated by an electronic-function-generator into gradual varying of cellular activities. It has been suggested that some master regulators displayed quantitative effects on cell fate decision, for instance, Oct/4 quantitatively influences differentiation, dedifferentiation or self-renewal of embryonic stem cells [26]; P53 quantitatively control cell fate decision between apoptosis and growth arrest [27]. Therefore, a synthetic circuit, which responds to various input programs and produced gradual output values, has the potential to be used as computer-aided cell fate controller. In addition, our 3-input circuit that responds to light and chemicals can be used to express a therapeutic gene at a specific place and time, meanwhile, exhibits minimized undesirable expression at other places, which would improve the safety of the therapy.

In natural biology systems, analog behaviors are common, for instance, stimulation of stress-responsive gene could be operated in an analog regime [28] and neurons perform both digital and analog information processing [29]. Moreover, the analog computing system has been demonstrated in *E. coli* [30–32]. As mentioned above, DAC is widely used in electronic engineering. A biological DAC-like module that combines multiple signals, and process the digital combinations of stimuli into graded output values for reconstruction of analog signal is needed to achieve sophisticated bio-computation functions, for example, programmable logic controller and reliable environmental sensor. In this study, by constructing a circuit that converts discrete input signals into varying of transcriptional activity of the output promoter, we attempted to explore the possibility that confers DAC merit, to a certain degree, to mammalian cells.

However, the output steps of our circuit were non-monotonic, therefore it may not act as a real DAC (Fig. 5 and Additional file 4: Figure S3). We hypothesize that modification of TCP sequence to increase its efficiency of rtTAm induction might be a potential way to increase the linearity of the output steps. Previous work has suggested the method to improve the function of TetR-inducing short peptides [20, 33]. Also, a further modification is required to expand the rationale to implement more inputs.

## Conclusion

We presented mammalian circuits that processed multi-input of blue light and chemical molecules. The 2-input circuit displayed blue light illumination dose-dependent shifting of Dox response threshold. The results suggested that increased expression of the upstream repressor (TetR) resulted in an increased activation threshold with similar basal expression level and dynamic range to that of the downstream TetR-repressible promoter. The 3-input circuit converted the sequence of blue light and two chemical molecules into varying of promoter activities over two orders of magnitude.

## Methods

### Construction of DNA plasmid

The details of DNA cloning are described in Additional file 1: Supplementary note.

### Cell culture, transient transfections, and generation of stable cell lines

Human Embryonic Kidney (HEK) 293 cells were obtained from the American Type Culture Collection (Manassas, VA), and were used in our previous study conducted by Dr. Zai Wang et al. [34]. The cells were grown in High Glucose Dulbecco’s modified Eagle’s medium (DMEM, Life Technologies, catalog number: 12800–017) supplemented with 10% FBS (Life Technologies, catalog number: 10270–106). The cells were sustained at 37 °C, in 5% CO_2_ environment. In this study, all transfections were conducted by using Lipofectamine 2000 Transfection Reagent (Life Technologies, catalog number: 11668–019). 24 h before DNA transfection, 0.5 × 10^6^ cells were seeded in each well of 6 well cell culture multiwell plate. On the day of DNA transfection, 5 μg DNA diluted in 500 μl Opti-MEM (Thermo Scientific, catalog number: 31985–070) mix with 10 μl Lipofectamine 2000 Transfection Reagent diluted in 500 μl Opti-MEM. The DNA-lipid complex was incubated at room temperature for 20 min, and then was added to the cells. To establish the desirable stable cell lines, 400 μg/ml Zeocin (InvivoGen, catalog number: ant-zn-1), 200 μg/ml Hygromycin B (Sigma-Aldrich, catalog number: H3274-50MG), and/or 2 μg/ml Puromycin (InvivoGen, catalog number: ant-pr-1) were correspondingly used to selected the cells for 10 to 14 days. Doxycycline (Sigma-Aldrich, catalog number 24390-14-5) and/or cumate solution (System Biosciences, catalog number: QM100A-1) were correspondingly used to treat the cells. For light-inducible expression, cells were sustained in a dark environment for 7 days before illumination with blue LED. To induce the LightOn controlled gene expression, the cells were illuminated for 24 h and then were put back to the dark environment for further 48 h, 24 h or 0 h culture before the measurements.

### Inducible gene expression

For light-inducible expression, cells were kept in darkness for 7 days before illumination with blue LED. Cells were exposed to blue light (1.25 W m^−2^) for 5 min daily to maintain a minimal level of TetR::mCherry::NLS for the suppression of downstream GFP, thereby ensuring cells remained in the same condition (GFP negative) before Dox induction. In the tuning activation threshold experiment, the cells were exposed to blue light for different time durations and then kept in darkness before treatment with various concentrations of Dox. For cumate-inducible expression, the inducer was added into the culture medium 72 h before measurement of gene expression.

### Fluorescence microscopy

The cells for microscopy images collection were grown in 60 mm dish, sustained in DMEM medium supplemented with 10% FBS, treated with the particular inducers, i.e., doxycycline, cumate and blue light. Images were taken at 72 h after induction started. Microscopy images were acquired on a Nikon TE2000-E inverted fluorescence microscope. The filter for fluorescent images of GFP was FITC (Ex 465–495 nm, Em 515–555 nm), exposure time was 3000 ms; for mCherry was Cy3 (Ex 530–560 nm, Em 573–648 nm), exposure time was 6000 ms; and for EYFP was YFP (Ex490-500, Em 520–560), exposure time was 3000 ms. Data processing was performed with software Image J.

### Flow cytometry

The flow cytometry analysis was carried out on BD LSR Fortessa Analyzer. Before the analysis, the cells were trypsinized using 0.25% trypsin–EDTA and then were centrifuged. 100,000 to 150,000 cells were suspended in 700 μl PBS supplemented with 1% FBS before loading onto the analyzer. GFP was measured with a 488 nm blue laser and an FITC (530/30 nm) emission filter, whereas mCherry was measured with a 561 nm yellow-green laser and a PE-Texas Red (610/20 nm) emission filter. FlowJo software was used to perform the data collection and processing.

### Luciferase assay

The Varioskan Flash Spectral Scanning Multimode Reader (Thermo Scientific) was used to measure the chemiluminescence catalyzed by luciferase. The Luciferase Assay System (Promega, catalog number E1500) was used for luciferase activity measurements. The measurements were carried out at 72 h after induction by following the kit instruments.

## Additional files

Additional file 1: Supplementary note. This note describes the details of DNA cloning; information of plasmids used in this study; information of oligonucleotides used in this study; nucleic acid sequence of CMV(tetO2) promoter and CMV5(CuO) promoter; amino acid sequence of rtTAm, TCP, and mCherry-TCP. (DOCX 30 kb)
Additional file 2: Figure S1. TCP fusion proteins induce rtTAm-dependent expression of GFP. We transfected HEK cells with mCherry, mCherry-TCP, or mCherry-NLS-TCP constitutive expression plasmids, respectively, also transfected TRE3G promoter controlled GFP expression plasmid to all the three groups of cells. We divided mCherry transfected cells into two dishes and treated one dish of the cells with 1 μg/ml Dox at 24 h after transfection. The images were collected at 72 h after transfection. (TIF 532 kb)
Additional file 3: Figure S2. Kinetics of the circuit. (A) Kinetics of the circuit responding to blue light illumination with constitutive rtTAm expression. The cells were illuminated by blue LED (1.25 W m^−2^) for 0 h, 5 h, 10 h, 20 h and 40 h, respectively. The data are presented as mean ± SD (*n* = 6). (B) Kinetics of the circuit responding to Dox with constitutive rtTAm expression. The cells were treated with 1 μg/ml of Dox for 0 h, 5 h, 10 h and 20 h, respectively. The data are presented as mean ± SD (*n* = 6). (C) Kinetics of the circuit responding to cumate with constitutive mCherry-TCP expression. The cells were treated with 30 μg/ml of cumate for 0 h, 5 h, 10 h, 20 h and 40 h, respectively. The data are presented as mean ± SD (*n* = 6). (TIF 1013 kb)
Additional file 4: Figure S3. (A) Scheme of the modified circuit with a conditional positive feedback loop in the third layer. We inserted a TetR and 3 × VP16 fusion (tTA) at downstream of the reporter luciferase. In the absence of Dox, the tTA binds to its own promoter and enhances the transcription from this promoter. (B) The output luciferase activities induced by different combinations of input signals. We used 10^5^ cells for the luciferase activity measurement. We repeated this experiments for three times. Data acquired in one of the three experiments were presented in mean ± SEM (*n* = 6). (TIF 465 kb)

## Abbreviations

CuO: p-cmt and p-cym operator site
CymR: p-cmt and p-cym operon repressor
Dox: Doxycycline
*E. coli*: *Escherichia coli*
EYFP: Enhanced yellow fluorescent protein
GAVPO: Gal4(65) and the smallest light-oxygen-voltage domain fusion protein
GFP: Green fluorescent protein
HEK 293 cells: Human embryonic kidney 293 cells
LED: Light-emitting diode
rtTA: Reverse tetracycline-controlled trans-activator
TCP: Tetracycline repressor co-repression peptides
tetO: Tetracycline operator sequence
TetR: Tetracycline repressor
TRE3G promoter: Third generation tetracycline-responsive element promoter
tTA: Tetracycline-controlled trans-activator
VP16: Herpes simplex virus protein VP16.
