# Supplementary material for: Synthetic circuits that process multiple light and chemical signal inputs
Source: BMC Syst Biol. 2017 Jan 19;11:5. doi: 10.1186/s12918-016-0384-y (PMC5244718; doi:10.1186/s12918-016-0384-y)
Supplement: Additional file 1: — Supplementary note. This note describes the details of DNA cloning; information of plasmids used in this study; information of oligonucleotides used in this study; nucleic acid sequence of CMV(tetO2) promoter and CMV5(CuO) promoter; amino acid sequence of rtTAm, TCP, and mCherry-TCP. (DOCX 30 kb) [file 12918_2016_384_MOESM1_ESM.docx]

**Synthetic circuits that process multiple light and chemical signal inputs**

Lizhong Liu^1,2^, Wei Huang^4^ and Jian-Dong Huang^1,2,3, *^

^1^ School of Biomedical Sciences, Li Ka Shing Faculty of Medicine, University of Hong Kong, Pok Fu Lam, Hong Kong, PR China,

^2^ Shenzhen Institute of Research and Innovation, University of Hong Kong, Shenzhen 518057, PR China,

^3^ The Centre for Synthetic Biology Engineering Research, Shenzhen Institutes of Advanced Technology, Shenzhen 518055, PR China,

^4^ Department of Biology, Shenzhen Key Laboratory of Cell Microenvironment, South University of Science and Technology of China, Shenzhen, China

* To whom correspondence should be addressed. Tel: +852 3917 6810; Fax: +852 2855 1254; Email: [jdhuang@hku.hk](mailto:jdhuang@hku.hk)

E-mail addresses of co-authors: LZL, [liulz@hku.hk](mailto:liulz@hku.hk); WH, huangw@sustc.edu.cn

**Construction of plasmids**

We list the plasmids used in this study in Table S1

To construct mCherry-TCP fusion and mCherry-NLS-TCP fusion expressing vectors, we used PCR to insert TCP or NLS-TCP coding sequence into mCherry ORF. Specifically, we used forward primer XbaI-mCherry-F and reverse primer EcoRI-TCP1-mcherry-R to amplify mCherry coding sequence from the plasmid BBa_1712028 (iGEM collections). We obtained mCherry-TCP fusion fragment. On the other hand, we used forward primer XbaI-mCherry-F and reverse primer EcoRI-TCP1-mcherryNLS-R to amplify mCherry-NLS from the plasmid BBa_1712028, and obtained mCherry-NLS-TCP fusion fragment. We digested the two fragments with XbaI and EcoRI, and then inserted them into PUC19 vector, respectively, for sequencing. Next, we used XbaI and EcoRI to remove mCherry-TCP and mCherry-NLS-TCP from the relevant PUC19 derived vectors. Next, we inserted these two fragments into pcDNA3.1 vector through NheI and EcoRI sites.

To construct a rtTAm constitutive expression vector, we generated a reverse TetR variant that can perform conformational change upon TCP binding to its inducer-binding pockets. The new conformation of reverse TetR allows it to bind to its cognate DNA sequence. Specifically, we introduced five mutations, i.e., E15A-L17G-L25V-M59I-S92R-H93Y to the wild type TetR *^(^*[*^1^*](#_ENREF_1)*^)^*. We used Gibson Assembly Cloning Kit (NEB, catalog number E5510S) to assemble the four PCR fragments containing the desired mutations to obtain the reverse TetR variant. A TetR coding plasmid pcDNA6/TR (Life Technologies) was used as PCR temple. The relevant primers are listed in Table S2. Sequencing confirmed the mutations. Next, we inserted 3 × VP16 coding sequence into the ORF of this reverse TetR at the C-terminal NdeI site. We called this reverse TetR variant and 3×VP16 fusion as rtTAm, which was placed downstream of a CMV promoter.

We constructed two versions of rtTAm-responsible expression vectors, one is TRE3G promoter driving expression of hrGFP, the other is TRE3G promoter driving expression of luciferase. We cloned TRE3G promoter (Clontech) into pU5-hrGFP ([2](#_ENREF_2)) at KpnI and HindIII sites to replace the U5 promoter. We also inserted the TRE3G promoter into pGL3-Basic (Promega) at SmaI and NcoI sites. Then we cut out the TRE3G promoter-luciferase cassette by digesting the plasmid with NheI and SalI and inserted this cassette into pBX-023 ([3](#_ENREF_3)), obtained pBX-TRE3G-luciferase.

We combined light-inducible mCherry-TCP expression cassette and cumate-inducible rtTAm expression cassette into a single vector. We first cloned rtTAm from the pCMV-rtTAm vector and inserted it into QM521A (System Biosciences, Cumate-switch system) at NheI and NotI sites. Then, we replaced the coGFP gene in QM521A with an EYFP gene. After this modification, rtTAm and EYFP were integrated into one ORF linked by 2A peptide. Next, we digested the pCMVCuO5-rtTAm-2A-EYFP plasmid with sfiI and HpaI and treated the fragment with T4 DNA Polymerase (NEB, catalog number M0203S) to generate blunt ends. The prepared fragment was then inserted into pU5-mCherry-TCP at MfeI site. We digested pU5-mCherry-TCP with MfeI and treated the linearized plasmid with T4 DNA Polymerase and Alkaline Phosphatase, Calf Intestinal (NEB, catalog number M0290S) before ligated it with the pCMVCuO5-rtTAm-2A-EYFP fragment. Since it was a blunt ends ligation, these two cassettes could be either be “face-to-face” or “back-to-back” ligated. We chose a “back-to-back” connected version for the followed experiments.

We established a conditional positive feedback plasmid. We first generated a TetR and 3 × VP16 fusion by fused 3 × VP16 sequence at the C-terminal end of TetR (TAA was removed) by PCR. We called this fusion as tTA. Then we placed the tTA gene downstream of TRE3G promoter via BamHI and MfeI digestion.

**Table S1.** Key plasmids used in this study

| Vector | Description | Reference |
| --- | --- | --- |
| QM200PA  pBX-GAVPO-zeocin  pCMV-mCherry-TCP  pCMV-mCherry-NLS-TCP  pCMV-rtTAm  pTRE3G-hrGFP  pU5-mCherry-TCP  pBX-TRE3G-luciferase  pU5-mCherry-TCP-CMVCuO5-rtTAm-2A-EYFP  pTRE3G-tTA | CymR and Puromycin resistance gene are co-expressed by EF1a promoter  The coding sequence of GAVPO and Zeocin linked with 2A peptide. This cassette is flanked by 5’ and 3’terminal repeats of PiggyBac transposon and HS4 insulator sequence.  CMV promoter drives expression of mCherry-TCP fusion  CMV promoter drives expression of mCherry-NLS-TCP fusion  CMV promoter drives expression of reverse TetR variant and 3×VP16 fusion  TRE3G promoter drives expression of the humanized recombinant GFP (hrGFP)  GAVPO-responsible promoter U5 drives expression of mCherry-TCP fusion  TRE3G-promoter drives expression of luciferase. This cassette is flanked by 5’ and 3’terminal repeats of PiggyBac transposon and HS4 insulator sequence.  GAVPO-responsible promoter U5 drives expression of mCherry-TCP fusion, and CMVCuO5 promoter drives co-expression of rtTAm and EYFP. These two cassettes are combined into one plasmid. A hygromycin resistant gene driven by SV40 promoter is also encoded in this plasmid.  TRE3G promoter derives expression of TetR and 3×VP16 fusion | System Biosciences (SBI)  Unpublished data  This study  This study  This study  This study  This study  This study  This study  This study |

**Table S2.** Key oligonucleotides used in this study

| Name | Sequence | Use |
| --- | --- | --- |
| XbaI-mcherry-F  EcoRI-TCP1-mcherry-R  EcoRI-TCP1-mcherryNLS-R  RevTetR-SLIC-1F  RevTetR-SLIC-1R  RevTetR-SLIC-2F  RevTetR-SLIC-2R  RevTetR-SLIC-3F  RevTetR-SLIC-3R  RevTetR-SLIC-4F  RevTetR-SLIC-4R | TATGTCTAGAGCCACCATGGTGAGCAAGGGCGAGGAG  TGAATTCTTAGTTCCAGCTGGGCAGCAGGCGGGCCACGG  CCATGATGATCTTGCCGGTCTTGTACAGCTCGTCC  GTGAATTCAGTTCCAGCTGGGCAGCAGGCGGGCCACGG  CCATGATGATCTTGCCGGTTACCTTTCTCTTCTTTTT  CAAGCTGGCTAGCGTTTAAACTTAAGCTTGGTACCC  TCACACCTTCGATTCCGACCTCATTGCCCAGGGCTAATGC  CCCTGGGCAATGAGGTCGGAATCGAAGGTGTGACAACC  CTAAGATCTCAATGGCTAAGGCGTCGAGCA  TGCTCGACGCCTTAGCCATTGAGATCTTAGATAGGC  CTTTTGCTCCATCGCGATATCTTAGTAAAGC  GCTTTACTAAGATATCGCGATGGAGCAAAAG  AAACAAGTTCTGCTTTAATAAGATCTGAATTCC | mCherry cloning  Add TCP to mCherry  Add TCP to mCherry-NLS  Introduce E15A-L17G-L25V-  M59I-S92R-H93Y to TetR |

**Sequence information**

**CMV(tetO2) promoter**

TATA box 573-579 bp

2 × tetO 589-628 bp

1 GTTGACATTG ATTATTGACT AGTTATTAAT AGTAATCAAT TACGGGGTCA TTAGTTCATA GCCCATATAT GGAGTTCCGC GTTACATAAC TTACGGTAAA

CAACTGTAAC TAATAACTGA TCAATAATTA TCATTAGTTA ATGCCCCAGT AATCAAGTAT CGGGTATATA CCTCAAGGCG CAATGTATTG AATGCCATTT

101 TGGCCCGCCT GGCTGACCGC CCAACGACCC CCGCCCATTG ACGTCAATAA TGACGTATGT TCCCATAGTA ACGCCAATAG GGACTTTCCA TTGACGTCAA

ACCGGGCGGA CCGACTGGCG GGTTGCTGGG GGCGGGTAAC TGCAGTTATT ACTGCATACA AGGGTATCAT TGCGGTTATC CCTGAAAGGT AACTGCAGTT

201 TGGGTGGAGT ATTTACGGTA AACTGCCCAC TTGGCAGTAC ATCAAGTGTA TCATATGCCA AGTACGCCCC CTATTGACGT CAATGACGGT AAATGGCCCG

ACCCACCTCA TAAATGCCAT TTGACGGGTG AACCGTCATG TAGTTCACAT AGTATACGGT TCATGCGGGG GATAACTGCA GTTACTGCCA TTTACCGGGC

301 CCTGGCATTA TGCCCAGTAC ATGACCTTAT GGGACTTTCC TACTTGGCAG TACATCTACG TATTAGTCAT CGCTATTACC ATGGTGATGC GGTTTTGGCA

GGACCGTAAT ACGGGTCATG TACTGGAATA CCCTGAAAGG ATGAACCGTC ATGTAGATGC ATAATCAGTA GCGATAATGG TACCACTACG CCAAAACCGT

401 GTACATCAAT GGGCGTGGAT AGCGGTTTGA CTCACGGGGA TTTCCAAGTC TCCACCCCAT TGACGTCAAT GGGAGTTTGT TTTGGAACCA AAATCAACGG

CATGTAGTTA CCCGCACCTA TCGCCAAACT GAGTGCCCCT AAAGGTTCAG AGGTGGGGTA ACTGCAGTTA CCCTCAAACA AAACCTTGGT TTTAGTTGCC

501 GACTTTCCAA AATGTCGTAA CAACTCCGCC CCATTGACGC AAATGGGCGG TAGGCGTGTA CGGTGGGAGG TCTATATAAG CAGAGCTCTC CCTATCAGTG

CTGAAAGGTT TTACAGCATT GTTGAGGCGG GGTAACTGCG TTTACCCGCC ATCCGCACAT GCCACCCTCC AGATATATTC GTCTCGAGAG GGATAGTCAC

601 ATAGAGATCT CCCTATCAGT GATAGAGATC GTCGACGAGC TCGTTTAGTG AACCGTCAGA TCGCCTGGAG ACGCCATCCA CGCTGTTTTG ACCTCCATAG

TATCTCTAGA GGGATAGTCA CTATCTCTAG CAGCTGCTCG AGCAAATCAC TTGGCAGTCT AGCGGACCTC TGCGGTAGGT GCGACAAAAC TGGAGGTATC

701 AAGACACCGG GACCGATCCA GCCTCCG

TTCTGTGGCC CTGGCTAGGT CGGAGGC

**CMV5(CuO) promoter**

CuO element 594-620 bp

1 AGACTAGTTA TTAATAGTAA TCAATTACGG GGTCATTAGT TCATAGCCCA TATATGGAGT TCCGCGTTAC ATAACTTACG GTAAATGGCC CGCCTGGCTG

TCTGATCAAT AATTATCATT AGTTAATGCC CCAGTAATCA AGTATCGGGT ATATACCTCA AGGCGCAATG TATTGAATGC CATTTACCGG GCGGACCGAC

101 ACCGCCCAAC GACCCCCGCC CATTGACGTC AATAATGACG TATGTTCCCA TAGTAACGCC AATAGGGACT TTCCATTGAC GTCAATGGGT GGAGTATTTA

TGGCGGGTTG CTGGGGGCGG GTAACTGCAG TTATTACTGC ATACAAGGGT ATCATTGCGG TTATCCCTGA AAGGTAACTG CAGTTACCCA CCTCATAAAT

201 CGGTAAACTG CCCACTTGGC AGTACATCAA GTGTATCATA TGCCAAGTCC GCCCCCTATT GACGTCAATG ACGGTAAATG GCCCGCCTGG CATTATGCCC

GCCATTTGAC GGGTGAACCG TCATGTAGTT CACATAGTAT ACGGTTCAGG CGGGGGATAA CTGCAGTTAC TGCCATTTAC CGGGCGGACC GTAATACGGG

301 AGTACATGAC CTTACGGGAC TTTCCTACTT GGCAGTACAT CTACGTATTA GTCATCGCTA TTACCATGGT GATGCGGTTT TGGCAGTACA CCAATGGGCG

TCATGTACTG GAATGCCCTG AAAGGATGAA CCGTCATGTA GATGCATAAT CAGTAGCGAT AATGGTACCA CTACGCCAAA ACCGTCATGT GGTTACCCGC

401 TGGATAGCGG TTTGACTCAC GGGGATTTCC AAGTCTCCAC CCCATTGACG TCAATGGGAG TTTGTTTTGG CACCAAAATC AACGGGACTT TCCAAAATGT

ACCTATCGCC AAACTGAGTG CCCCTAAAGG TTCAGAGGTG GGGTAACTGC AGTTACCCTC AAACAAAACC GTGGTTTTAG TTGCCCTGAA AGGTTTTACA

501 CGTAATAACC CCGCCCCGTT GACGCAAATG GGCAAGCTTG CCGGGTCGAG GTAGGCGTGT ACGGTGGGAG GCCTATATAA GCAACCGGTA TAATACAAAC

GCATTATTGG GGCGGGGCAA CTGCGTTTAC CCGTTCGAAC GGCCCAGCTC CATCCGCACA TGCCACCCTC CGGATATATT CGTTGGCCAT ATTATGTTTG

601 AGACCAGATT GTCTGTTTGT TACCGGTGTT TAGTGAACCG GGCGCGCCTC ATATCGCCTG GAGACGCCAT CCACGCTGTT TTGACCTCCA TAGAAGACAC

TCTGGTCTAA CAGACAAACA ATGGCCACAA ATCACTTGGC CCGCGCGGAG TATAGCGGAC CTCTGCGGTA GGTGCGACAA AACTGGAGGT ATCTTCTGTG

701 CGGGACCGAT CCAGCCTCCG CGGTCACTCT CTTCCGCATC GCTGTCTGCG AGGGCCAGCT GTTGGGCTCG CGGTTGAGGA CAAACTCTTC GCGGTCTTTC

GCCCTGGCTA GGTCGGAGGC GCCAGTGAGA GAAGGCGTAG CGACAGACGC TCCCGGTCGA CAACCCGAGC GCCAACTCCT GTTTGAGAAG CGCCAGAAAG

801 CAGTACTCTT GGATCGGAAA CCCGTCGGCC TCCGAACGGT ACTCCGCCAC CGAGGGACCT GAGCCAGTCC GCATCGACCG GATCGGAAAA CCTCTCGAGA

GTCATGAGAA CCTAGCCTTT GGGCAGCCGG AGGCTTGCCA TGAGGCGGTG GCTCCCTGGA CTCGGTCAGG CGTAGCTGGC CTAGCCTTTT GGAGAGCTCT

901 AAGGCGTCTA ACCAGTCACA GTCGCAAGGT AGGCTGAGCA CCGTGGCGGG CGGCAGCGGG TGGCGGTCGG GGTTGTTTCT GGCGGAGGTG CTGCTGATGA

TTCCGCAGAT TGGTCAGTGT CAGCGTTCCA TCCGACTCGT GGCACCGCCC GCCGTCGCCC ACCGCCAGCC CCAACAAAGA CCGCCTCCAC GACGACTACT

1001 TGTAATTAAA GTAGGCGGTC TTGAGCCGGC GGATGGTCGA GGTGAGGTGT GGCAGGCTTG AGATCCAGCT GTTGGGGTGA GTACTCCCTC TCAAAAGCGG

ACATTAATTT CATCCGCCAG AACTCGGCCG CCTACCAGCT CCACTCCACA CCGTCCGAAC TCTAGGTCGA CAACCCCACT CATGAGGGAG AGTTTTCGCC

1101 GCATGACTTC TGCGCTAAGA TTGTCAGTTT CCAAAAACGA GGAGGATTTG ATATTCACCT GGCCCGATCT GGCCATACAC TTGAGTGACA ATGACATCCA

CGTACTGAAG ACGCGATTCT AACAGTCAAA GGTTTTTGCT CCTCCTAAAC TATAAGTGGA CCGGGCTAGA CCGGTATGTG AACTCACTGT TACTGTAGGT

1201 CTTTGCCTTT CTCTCCACAG GTGTCCACTC CCAGGTCCAA GTTT

GAAACGGAAA GAGAGGTGTC CACAGGTGAG GGTCCAGGTT CAAA

**Amino acid sequence of rtTAm**

Reverse tetR variant 1-206 aa

3 × VP16 207-249 aa

MSRLDKSKVINSALALGNEVGIEGVTTRKLAQKLGVEQPTLYWHVKNKRALLDALAIEILDRHHTHFCPLEGESWQDFLRNNAKSFRCALLRYRDGAKVHLGTRPTEKQYETLENQLAFLCQQGFSLENALYALSAVGHFTLGCVLEDQEHQVAKEERETPTTDSMPPLLRQAIELFDHQGAEPAFLFGLELIICGLEKQLKCESGGPTDALDDFDLDMLPADALDDFDLDMLPADALDDFDLDMLPG

**Amino acid sequence of TCP**

TGKIIMAVARLLPSWN

**Amino acid sequence of mCherry-TCP**

MVSKGEEDNMAIIKEFMRFKVHMEGSVNGHEFEIEGEGEGRPYEGTQTAKLKVTKGGPLPFAWDILSPQFMYGSKAYVKHPADIPDYLKLSFPEGFKWERVMNFEDGGVVTVTQDSSLQDGEFIYKVKLRGTNFPSDGPVMQKKTMGWEASSERMYPEDGALKGEIKQRLKLKDGGHYDAEVKTTYKAKKPVQLPGAYNVNIKLDITSHNEDYTIVEQYERAEGRHSTGGMDELYKTGKIIMAVARLLPSWN

**REFERENCES**

1. Goeke, D., Kaspar, D., Stoeckle, C., Grubmüller, S., Berens, C., Klotzsche, M., and Hillen, W. Short peptides act as inducers, anti-Inducers and corepressors of tet repressor. J. Mol. Biol. 2012;*416*:33-45.

2. Wang, X., Chen, X. J., and Yang, Y. Spatiotemporal control of gene expression by a light-switchable transgene system. Nat Methods. 2012;*9*:266-269.

3. Lu, X. B., and Huang, W. PiggyBac mediated multiplex gene transfer in mouse embryonic stem cell. PloS one. 2014;9:e115072.
