# Supplementary figures and images for: Synthetic circuits that process multiple light and chemical signal inputs
Source: BMC Syst Biol. 2017 Jan 19;11:5. doi: 10.1186/s12918-016-0384-y (PMC5244718; doi:10.1186/s12918-016-0384-y)

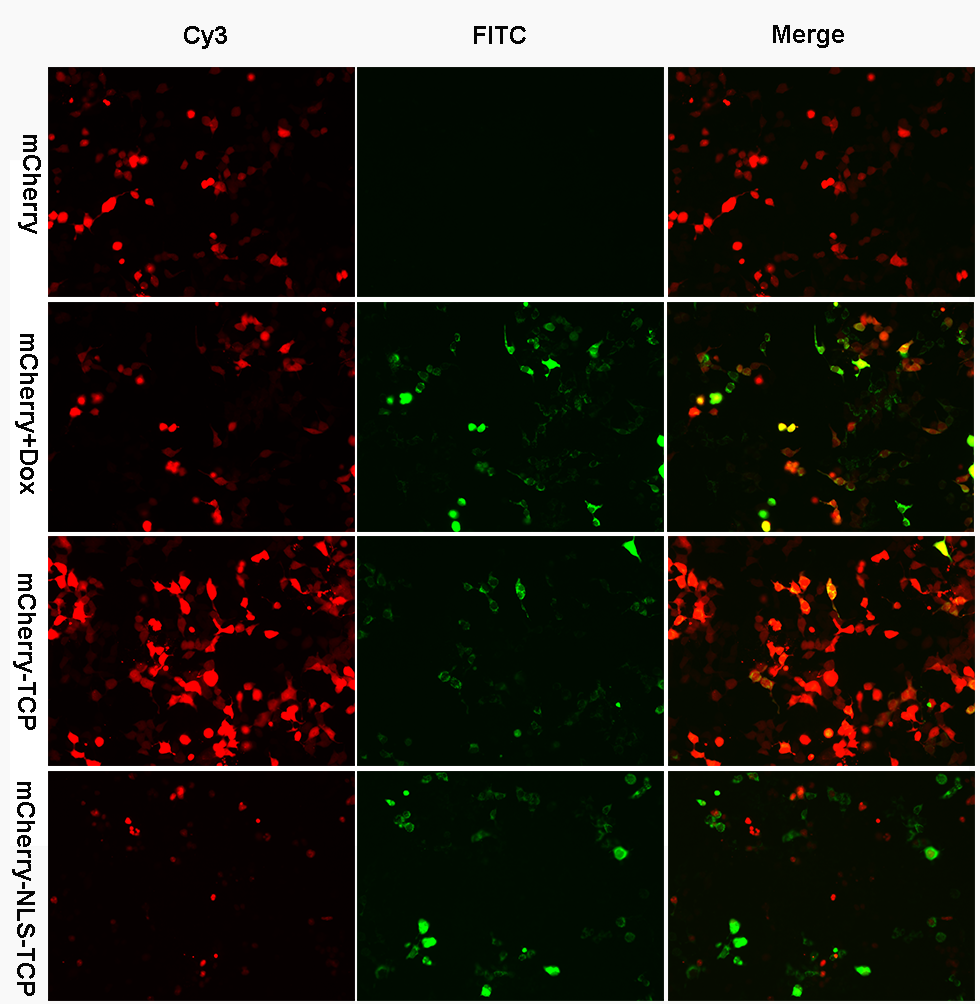

Supplement: Additional file 2: Figure S1. — TCP fusion proteins induce rtTAm-dependent expression of GFP. We transfected HEK cells with mCherry, mCherry-TCP, or mCherry-NLS-TCP constitutive expression plasmids, respectively, also transfected TRE3G promoter controlled GFP expression plasmid to all the three groups of cells. We divided mCherry transfected cells into two dishes and treated one dish of the cells with 1 μg/ml Dox at 24 h after transfection. The images were collected at 72 h after transfection. (TIF 532 kb) [file 12918_2016_384_MOESM2_ESM.tif]

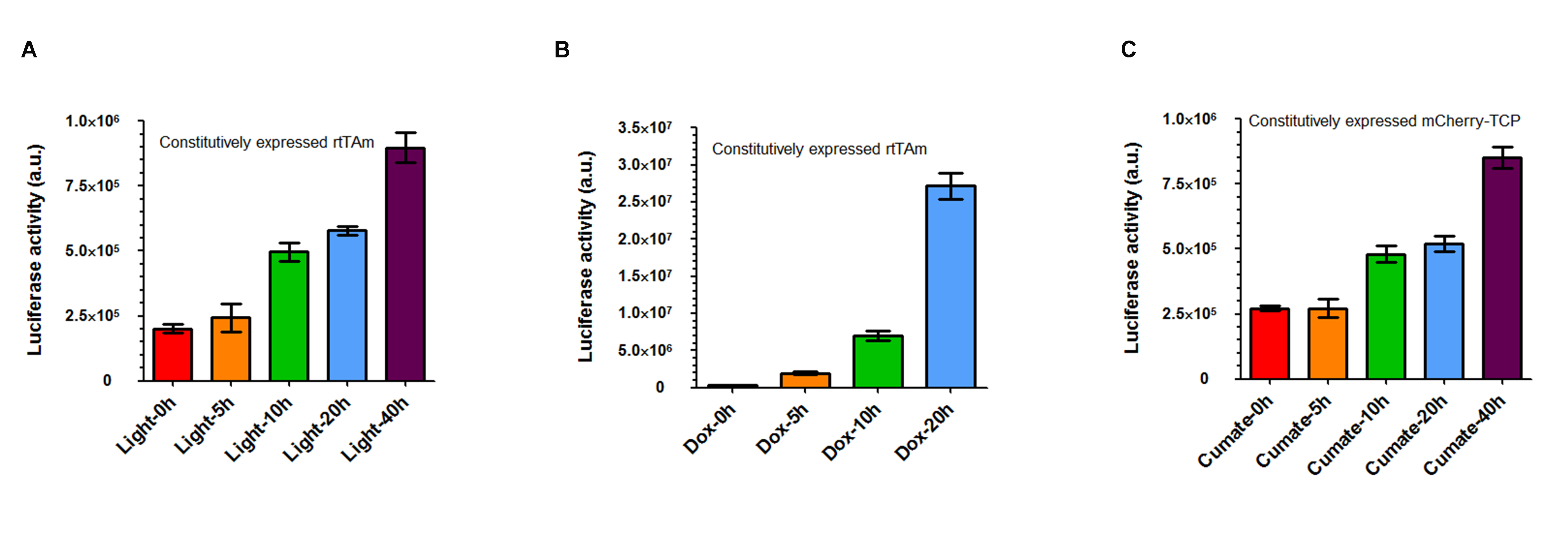

Supplement: Additional file 3: Figure S2. — Kinetics of the circuit. (A) Kinetics of the circuit responding to blue light illumination with constitutive rtTAm expression. The cells were illuminated by blue LED (1.25 W m−2) for 0 h, 5 h, 10 h, 20 h and 40 h, respectively. The data are presented as mean ± SD (n = 6). (B) Kinetics of the circuit responding to Dox with constitutive rtTAm expression. The cells were treated with 1 μg/ml of Dox for 0 h, 5 h, 10 h and 20 h, respectively. The data are presented as mean ± SD (n = 6). (C) Kinetics of the circuit responding to cumate with constitutive mCherry-TCP expression. The cells were treated with 30 μg/ml of cumate for 0 h, 5 h, 10 h, 20 h and 40 h, respectively. The data are presented as mean ± SD (n = 6). (TIF 1013 kb) [file 12918_2016_384_MOESM3_ESM.tif]

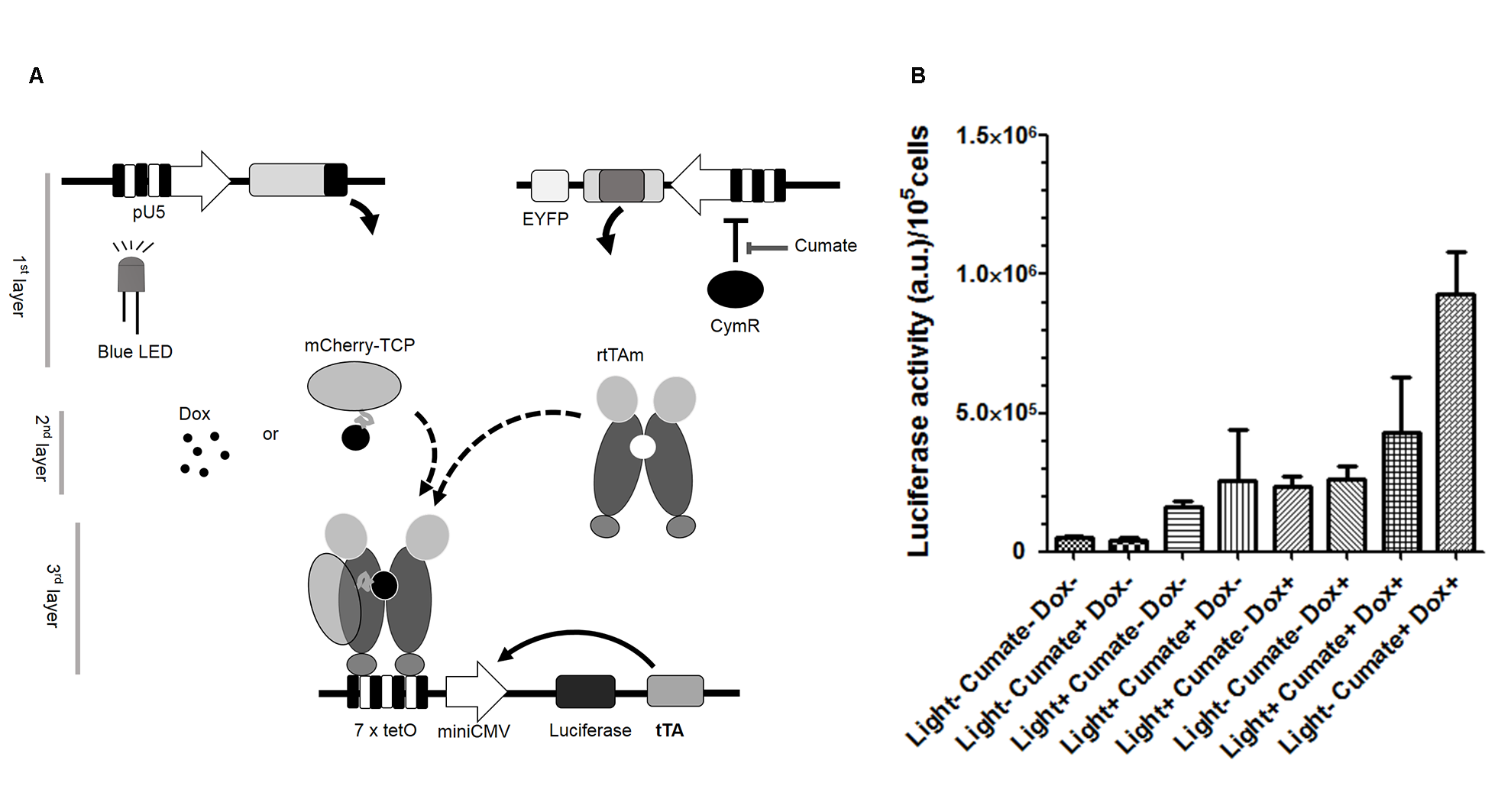

Supplement: Additional file 4: Figure S3. — (A) Scheme of the modified circuit with a conditional positive feedback loop in the third layer. We inserted a TetR and 3 × VP16 fusion (tTA) at downstream of the reporter luciferase. In the absence of Dox, the tTA binds to its own promoter and enhances the transcription from this promoter. (B) The output luciferase activities induced by different combinations of input signals. We used 105 cells for the luciferase activity measurement. We repeated this experiments for three times. Data acquired in one of the three experiments were presented in mean ± SEM (n = 6). (TIF 465 kb) [file 12918_2016_384_MOESM4_ESM.tif]
